# Magma reservoir dynamics at Toba caldera, Indonesia, recorded by oxygen isotope zoning in quartz

**DOI:** 10.1038/srep40624

**Published:** 2017-01-25

**Authors:** David A. Budd, Valentin R. Troll, Frances M. Deegan, Ester M. Jolis, Victoria C. Smith, Martin J. Whitehouse, Chris Harris, Carmela Freda, David R. Hilton, Sæmundur A. Halldórsson, Ilya N. Bindeman

**Affiliations:** 1 Department of Earth Sciences, CEMPEG, Uppsala University, Sweden; 2 Istituto Nazionale di Geofisica e Vulcanologia, Rome, Italy; 3 Department of Geosciences, Swedish Museum of Natural History, Stockholm, Sweden; 4 Research Laboratory for Archaeology and the History of Art, University of Oxford, Oxford, UK; 5 Department of Geological Sciences, University of Cape Town, South Africa; 6 Scripps Institution of Oceanography, University of California, San Diego, USA; 7 Institute of Earth Sciences, University of Iceland, Reykjavik, Iceland; 8 Department of Geological Sciences, University of Oregon, Oregon, USA

## Abstract

Quartz is a common phase in high-silica igneous rocks and is resistant to post-eruptive alteration, thus offering a reliable record of magmatic processes in silicic magma systems. Here we employ the 75 ka Toba super-eruption as a case study to show that quartz can resolve late-stage temporal changes in magmatic δ^18^O values. Overall, Toba quartz crystals exhibit comparatively high δ^18^O values, up to 10.2‰, due to magma residence within, and assimilation of, local granite basement. However, some 40% of the analysed quartz crystals display a decrease in δ^18^O values in outermost growth zones compared to their cores, with values as low as 6.7‰ (maximum ∆_core−rim_ = 1.8‰). These lower values are consistent with the limited zircon record available for Toba, and the crystallisation history of Toba quartz traces an influx of a low-δ^18^O component into the magma reservoir just prior to eruption. Here we argue that this late-stage low-δ^18^O component is derived from hydrothermally-altered roof material. Our study demonstrates that quartz isotope stratigraphy can resolve magmatic events that may remain undetected by whole-rock or zircon isotope studies, and that assimilation of altered roof material may represent a viable eruption trigger in large Toba-style magmatic systems.

The recent advent of micro-analytical techniques has dramatically increased our capabilities of crystal isotope fingerprinting with notable examples including micro-drilling associated with thermal ionisation mass spectrometry (TIMS), laser ablation multi-collector inductively coupled plasma mass spectrometry (LA-MC-ICPMS), and secondary ionisation mass spectrometry (SIMS) (e.g., ref. 1). These tools permit determination of isotope ratios on a sub-crystal scale, allowing the analysis of small sample volumes and thus production of high spatial resolution data within and between individual crystal growth zones (e.g., refs 2, 3, 4).

Crystal-scale oxygen isotope analysis is now routinely carried out using the SIMS technique^2^,^3^,^4^,^5^. For example, the oxygen isotopic composition of zircon is a robust source of information on the crystallisation environment^3^,^4^,^6^ and, as zircon also lends itself to U-Pb dating, it can help to identify and place constraints on the timing of geological events (e.g., refs 6 and 7). Quartz is a common mineral phase in high-silica metamorphic and magmatic rocks and, like zircon, is largely resistant to alteration and retains its magmatic δ^18^O values post-eruption^8^. Unlike zircon, however, quartz can grow to relatively large crystal sizes (e.g., up to 2 mm across in this study), which permits spatially detailed isotopic profiling with SIMS. This level of detail is more challenging to obtain in zircon as crystal size is typically ≤200 μm in most volcanic and metamorphic rocks, which limits intra-crystal analyses to usually a single core and perhaps one or two rim analyses (e.g., refs 2 and 9). Quartz may therefore be especially useful in certain high-silica magmatic settings, such as the Toba caldera in Indonesia, whose erupted products host plentiful quartz phenocrysts. Moreover, because oxygen diffusion in quartz is >10^4^ times faster than in zircon at magmatic temperatures^10^,^11^, quartz can potentially provide insight into shorter timescale magmatic processes compared to zircon. Therefore, isotopic changes preserved between the inner and outer rims of quartz crystals may correspond to relatively short timescale magmatic processes e.g., on the order of hundreds of years prior to eruption (cf. ref. 12). Indeed, Matthews *et al*. (ref. 12) previously applied Ti-diffusion chronometry to quartz crystals from the Young Toba Tuff (YTT) and suggested that short, episodic magma recharge events on a ca. 100-year timescale were important in priming the large-volume YTT magma reservoir for eruption. In the current paper, we present detailed SIMS oxygen isotope analysis of quartz crystals from the YTT, including several of the quartz crystals analysed by ref. 12 for diffusion chronometry to i) test for magma recharge, mixing, and pre-eruptive crustal recycling in the YTT reservoir as recorded in the crystal-scale oxygen isotope record, and ii) assess the utility of oxygen isotope stratigraphy in quartz as a tracer of pre-eruptive magmatic processes in large-volume rhyolitic systems.

## Geological setting

**Figure 1:**
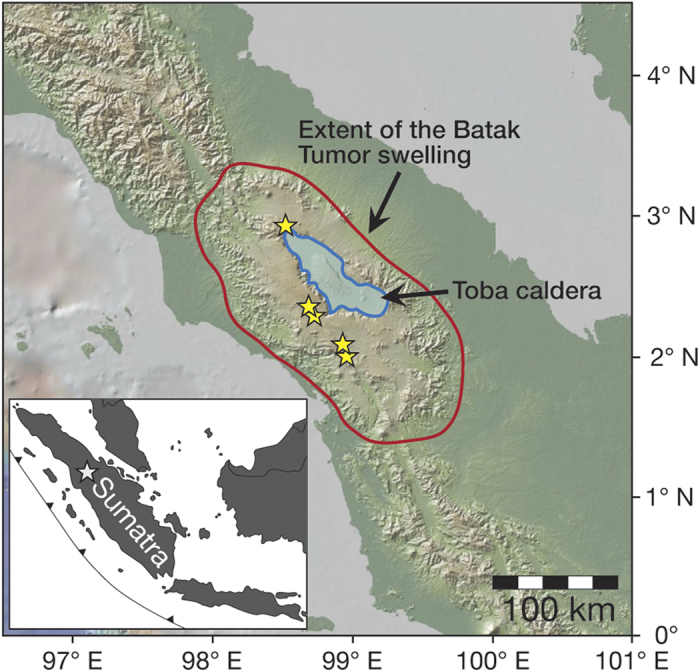
Location of study area. Map of northern Sumatra (adapted from GeoMapApp; www.geomapapp.org). The red ellipse designates the crustal swell of the Batak Tumor^15^, upon which the Toba caldera sits (in blue). The topographic swell is believed to result from upward pressure from the buoyant Toba magma system^15^. Yellow stars indicate sampling sites. Inset: Map of western Indonesia, with Toba volcano marked as white star (modified after ref. 62).

The Toba caldera, north Sumatra (Fig. 1), is thought to originate from subduction of the Investigator Ridge transform fault sited on the Indo-Australian Plate beneath the Sunda Trench, leading to formation of a deep crustal hot zone^13^,^14^ that feeds the Toba volcano. The Toba system has produced four major explosive eruptions over the last 1.2 million years^15^,^16^, the oldest being the 35 km^3^ Harrangoal Dacite Tuff (HDT). This event was followed by the quartz-rich 1,000 km^3^ Old Toba Tuff (OTT) ~0.79 Ma^17^, and the 60 km^3^ rhyolitic Middle Toba Tuff (MTT) ~0.5 Ma^18^. Finally, the cataclysmic 2,800 km^3^ rhyolitic Youngest Toba Tuff (YTT) erupted ca. 75 ka^19^. The YTT deposits extend over 20,000 km^2^ on Sumatra and are recorded as far as 2,000 km away (e.g. in India)^20^. The Toba super-eruption is the largest and best-known of the late Quaternary, and was speculated to have brought mankind to near extinction^21^.

**Figure 2:**
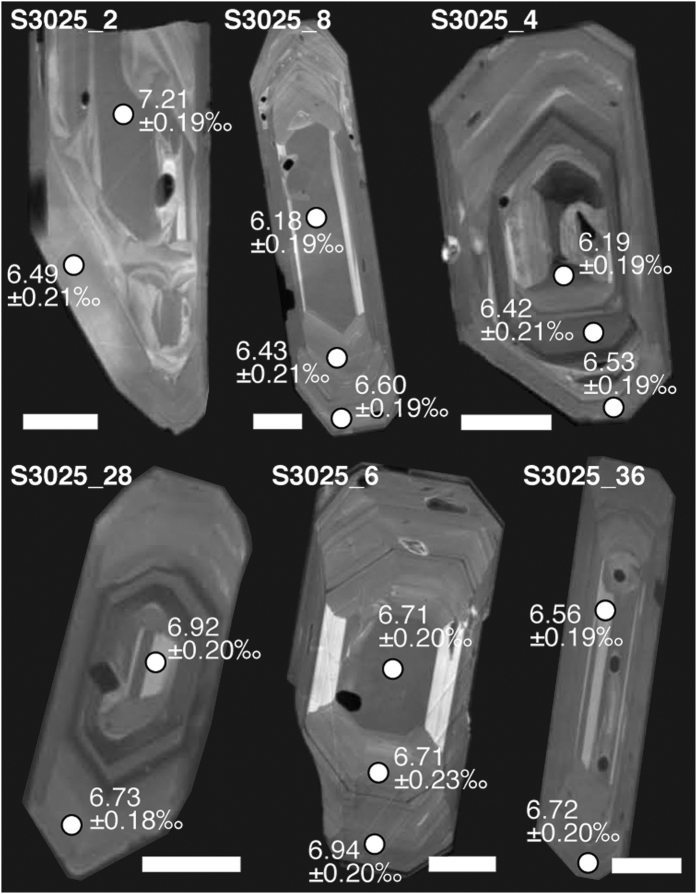
Textural and oxygen isotopic zoning in YTT zircon. Cathodoluminescence (CL) images of YTT zircons are shown with associated Secondary Ionisation Mass Spectrometry (SIMS) δ^18^O analysis spots. The data reveal a complex system with variable δ^18^O inputs. Notably, analyses are limited to two to three SIMS spots per crystal due to the relatively small crystal size of zircon. Error bars = 1σ and white scale bars = 50 μm. Data from ref. 9.

High-silica caldera systems like Toba (Indonesia), Yellowstone (USA) and Taupo (New Zealand) produce some of the largest volume eruptions on Earth^22^. Recent work on magma petrogenesis of the Yellowstone hot spot track, for example, has revealed oxygen isotope diversity in zircon that includes both high- and low-δ^18^O values relative to ‘normal’ rhyolites^9^. For instance, the higher δ^18^O values in Yellowstone zircon are thought to represent assimilation of unaltered crust by ascending magmas, whereas the lower end of the δ^18^O_zircon_ spectrum has been attributed to recycling of hydrothermally-altered (low-δ^18^O) rhyolitic material into the Yellowstone magma system. Similar intra-crystalline isotopic diversity is observed in zircon from other high-silica magmatic systems worldwide, including Toba, where comparable processes are hypothesised to have been in operation (Fig. 2 and ref. 9). To fully resolve the complexity of the Toba crystal record, however, isotopic heterogeneities need to be resolved in more detail. We therefore use Toba as a case-study to assess the utility of quartz as a tracer of pre-eruptive magmatic processes, especially since Toba eruptive rocks are particularly quartz rich^23^,^24^. Furthermore, widespread low-δ^18^O crust is not expected at Toba’s latitude^25^, meaning that we can also test for the role of late-stage additions of volcanically produced high-temperature hydrothermally-altered crust in driving large silicic eruptions.

## Petrography and geochemistry

The YTT samples used in this study are from stratigraphically constrained tuff units exposed along the caldera margin and from outcrops surrounding the Toba caldera. Bulk tuff and pumice samples exhibit no signs of alteration, and have a mineral assemblage comprising quartz, plagioclase, sanidine, biotite, amphibole, and minor pyroxene, zircon and oxides (cf. ref. 23). Bulk tuff and pumice samples have SiO_2_ contents between 71–73 wt.% (Supplementary Table 1), which fall within the mid-range of pumice compositions previously reported for the YTT (SiO_2_ = 68–77 wt.%) (e.g., ref. 23).

To provide comparative data for our SIMS approach, we also performed oxygen isotope analyses on YTT whole-rock tuff and pumice clasts by conventional fluorination and on separated crystal phases by bulk crystal laser fluorination (LF) (see Methods for details of analytical procedures). The whole-rock data have δ^18^O values between 8.2 and 9.9‰ (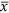 = 9.0 ± 0.15‰, 1σ, n = 11; Supplementary Table 2), whereas the older HDT (1.2 My; 61 wt.% SiO_2_) has a δ^18^O_whole-rock_ value of 8.6‰ (n = 1). The YTT bulk crystal analyses by LF give δ^18^O values of 8.2 to 9.3‰ for quartz (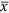 = 8.9 ± 0.15‰, 1σ, n = 13), 7.6 to 8.1‰ for feldspar (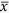 = 7.8 ± 0.15‰, 1σ, n = 6), 6.8 to 8.9‰ for biotite (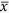 = 7.2 ± 0.15‰, 1σ, n = 8), and 5.5 to 6.4‰ for amphibole (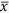 = 5.9 ± 0.15‰, 1σ, n = 4). In comparison, the δ^18^O_quartz_ values from basement granitoids near Toba range from 6.4 to 9.5‰ (±0.15‰, 1σ, n = 5).

Quartz crystals in the YTT are typically fragmented due to the high explosivity of the eruption^26^, but a record of multiple dissolution and reprecipitation events can nevertheless be discerned (e.g., ref. 24). To resolve spatial δ^18^O zonation within individual quartz crystals, we coupled cathodoluminescence (CL) and SIMS oxygen isotope analyses of 15 quartz crystals from pumice (reflecting pre-eruptive magma batches) and from bulk tuff (encapsulating mixed material on eruption). We present a total of 191 individual SIMS spot analyses that define a range of δ^18^O_quartz_ from 6.7 to 10.2‰ (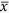 = 8.8 ± 0.4‰, 1σ; Supplementary Table 3).

In order to assess the meaningfulness of our data in a wider Toba context, we furthermore analysed two clinopyroxene samples from the 1.2 Ma HDT for their ^3^He/^4^He ratios. We note that the YTT is virtually devoid of pyroxene (see ref. 23) and hence it was unfeasible to extract sufficient crystals for helium isotope analysis. The ^3^He/^4^He values obtained (0.67 R_A_ and 1.76 R_A_, where R_A_ = air ^3^He/^4^He) are lower than typical mantle values (8 ± 1 R_A_) or contemporary geothermal fluid values at Toba (6.6 R_A_; ref. 27), and instead approach crustal values (<0.5 R_A_). Moreover, one whole-rock YTT sample was also analysed for Sr-Nd isotopes and yielded an ^87^Sr/^86^Sr value of 0.714033 (±13) and a ^143^Nd/^144^Nd value of 0.512113 (±13). These radiogenic isotope data, although few in number, are consistent with previous studies that used radiogenic isotopes and which suggested that substantial crustal material was indeed involved in YTT petrogenesis^28^,^29^.

## Resolving crustal differentiation processes at Toba

**Figure 3:**
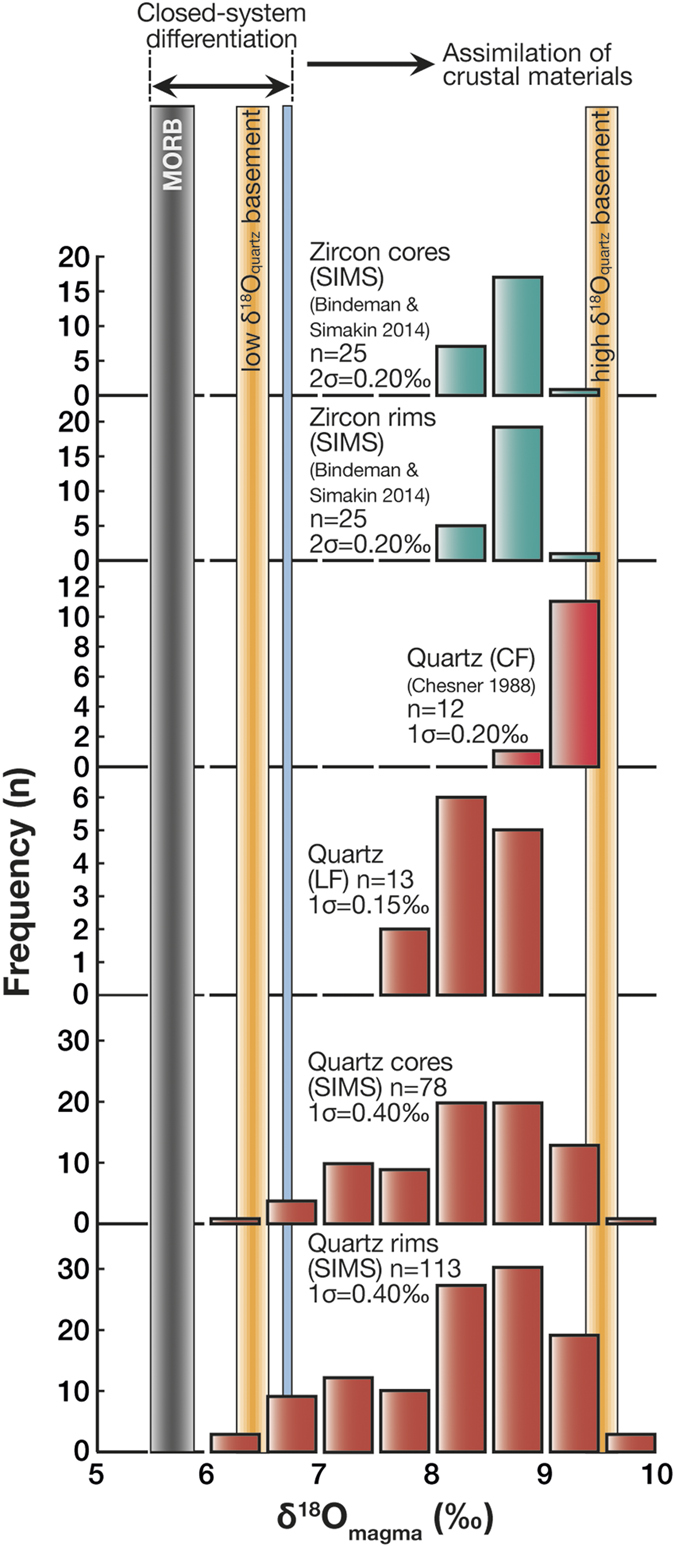
δ^18^O magma values for Toba. The δ^18^O quartz data are converted to equilibrium magma values assuming Δ^18^O_quartz-rhyolite_ = 0.5‰ at a temperature interval of 750–780 °C (ref. 31). SIMS zircon data are converted to magma values using the fractionation factor of ref. 63 and are then compared with LF crystal data (this study), conventional fluorination crystal data (CF; ref. 32), and available zircon SIMS data from ref. 9. The grey bar marks the δ^18^O value of a typical Sunda arc mantle-type basalt composition (MORB or I-MORB), and the blue bar denotes the highest δ^18^O values that can be obtained by closed-system crystal fractionation from a Sunda arc basaltic parent magma^33^,^34^,^35^. The orange bars denote the δ^18^O values of the crustal materials in the region. Our new quartz data overlap with the published zircon and quartz data from the YTT^9^,^32^, but additionally document isotopic excursions towards both higher and lower δ^18^O values than previously reported. Abbreviations in figure: CF, Conventional Fluorination; LF, Laser Fluorination; MORB, Mid-Ocean Ridge Basalt; SIMS, Secondary Ionisation Mass Spectrometry.

As is often the case in high-resolution analytical approaches^2^,^30^, our new SIMS data extend the established δ^18^O range for the YTT crystal data towards both higher and lower values, with an overall range of 3.5‰ for the full set of YTT quartz crystals (Fig. 3). Employing a crystal-melt fractionation factor (Δ^18^O_quartz-rhyolite_) of 0.5‰ (e.g., ref. 31), the δ^18^O value of the Toba magma(s) can be calculated from the derived crystal data. The average calculated δ^18^O_magma_ value is 8.3 ± 0.4‰ (n = 191, 1σ) for SIMS data and 8.4 ± 0.2‰ (n = 13, 1σ) for the LF data. The datasets show significant overlap, thus ruling out standardisation bias. Moreover, they overlap with calculated δ^18^O_magma_ values determined using YTT zircon data from the literature^9^ (average δ^18^O_magma_ value = 8.6 ± 0.2‰; n = 50, 2σ) (Fig. 3). We note that the bulk crystal conventional fluorination quartz data (ref. 32; data obtained pre-1990) produce a slightly higher average δ^18^O_magma_ value of 9.1 ± 0.2‰ (n = 12, 1σ) compared to our bulk quartz data (Fig. 3). This is likely due to our use of the more modern laser fluorination technique that provides more accurate results. Nevertheless, it is important to note that all YTT δ^18^O quartz and corresponding magma values are elevated relative to primitive Sunda arc basalt, which is expected to have δ^18^O values between 5.5 and 6.1‰ (refs 33, 34, 35), and ~6.7‰ for its closed-system rhyolitic differentiates (ref. 36). Therefore, we suggest that values ≥7‰ in the YTT quartz crystals indicate addition of an external high δ^18^O component.

**Figure 4:**
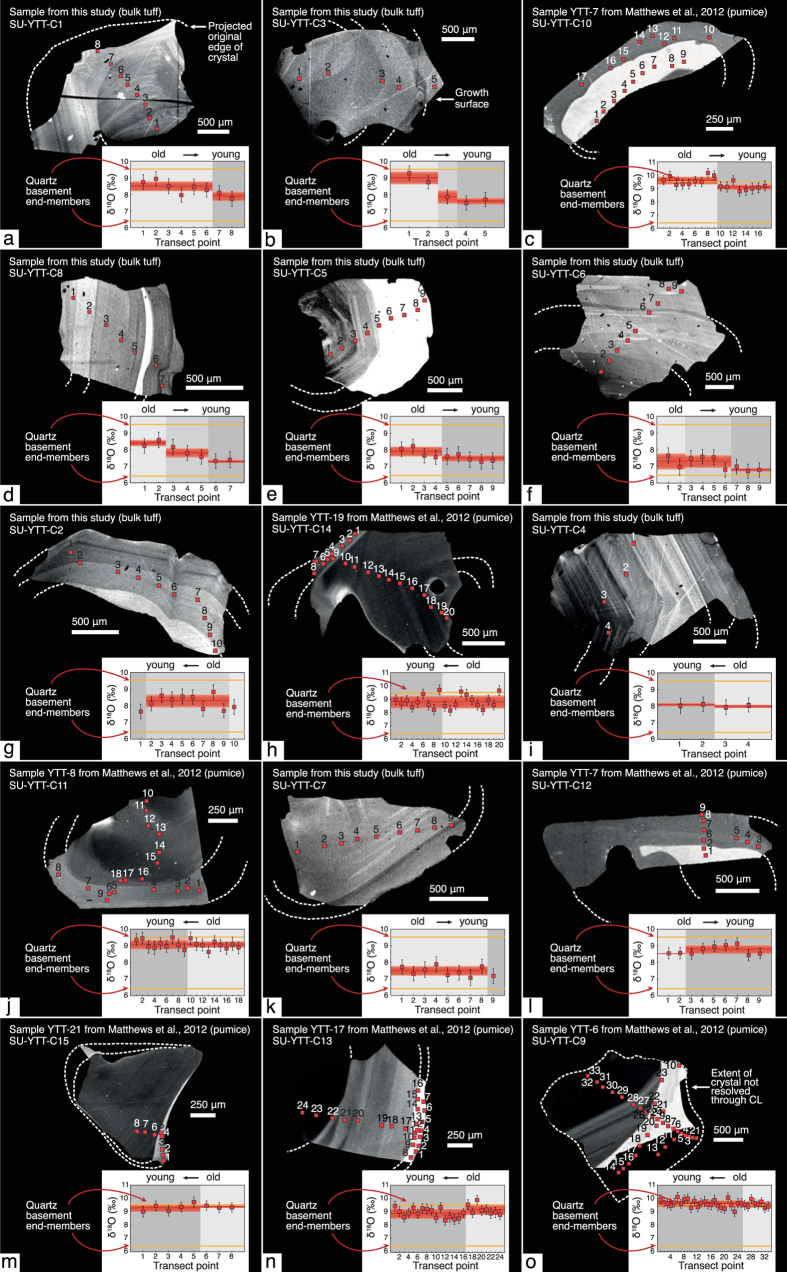
CL images and measured SIMS δ^18^O traverses across selected quartz crystals from the YTT. Grey shading on the Cathodoluminescence (CL) images defines distinct textural and compositional domains. Numbered red squares on the CL images correspond to analysis spots on the inset δ^18^O plots. Insets: Grey shading on graphs indicates textural domains and horizontal red bars indicate individual δ^18^O zone averages. Orange bars indicate high- and low-δ^18^O_quartz_ from basement granitoids of the Toba region. Error bars = 1σ. Crystals (**a**–**f**) (part 1) display an overall core to rim decrease in δ^18^O values, while crystals (**g**–**o**) (part 2) show no significant core to rim variation in δ^18^O value. Individual analysis points that deviate from the crystal average in (**g**–**o**) are considered outliers, and are potentially due to small inclusions of foreign material in the analysis.

Turning our attention to the single crystal data available prior to this study, there is evidence for intra-crystal isotopic heterogeneity from a reconnaissance investigation of zircon from the YTT (beyond 1σ; maximum ∆^18^O_core−rim_ = 0.72‰; Fig. 2 and ref. 9) but more data are required to fully resolve the issue of intra- and inter-crystal variability at Toba. Here, we take the opportunity to better resolve isotopic diversity within and between single crystal grains of our extensive sample suite, with the added advantage that our quartz crystals are larger than the zircon grains of the reconnaissance study^9^, thus permitting more detailed δ^18^O profiling through the crystals. Further, by adopting the SIMS approach, we overcome the blending effect that is usually associated with analysis of bulk rock or bulk crystals (cf. ref. 32). Indeed, individual YTT quartz crystals demonstrate core to rim variation of up to 1.8‰, and, importantly, ~40% of the analysed quartz crystals show a significant decrease (beyond 1σ) in their δ^18^O values from their cores to their outer growth zones (Fig. 4). The remaining ~60% of the analysed quartz crystals display limited resolvable core to rim variation in δ^18^O values along the analysis profiles. The possibility to conduct a large number of intra-crystal analyses in single quartz grains therefore allows us to record isotopic changes over small length- and hence time-scales, and so counters the averaging effect of whole-grain crystal data or spatially-limited intra-crystal traverses, as is often carried out for co-magmatic zircon (cf. refs 37 and 38) (Fig. 2).

**Figure 5:**
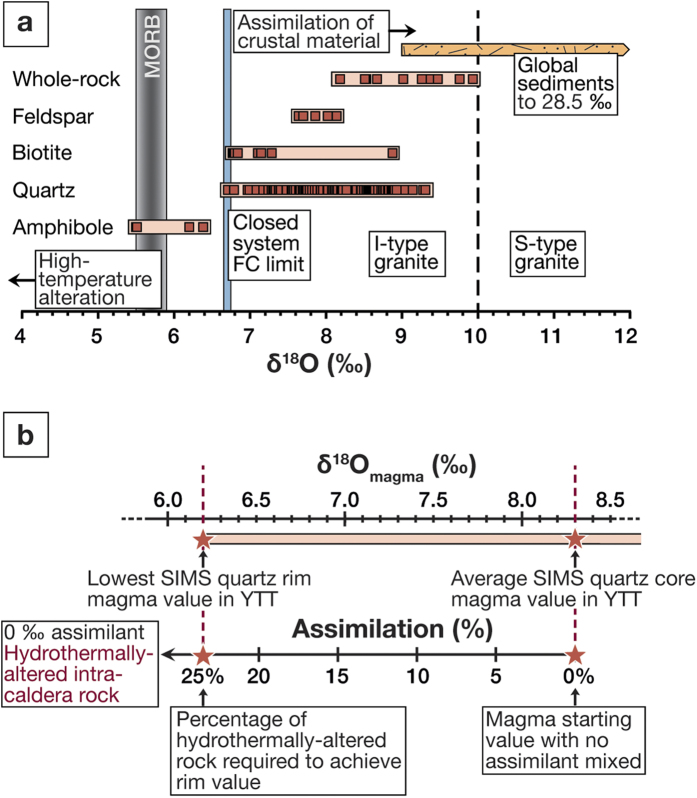
Magma diversification at Toba. (**a**) Summary figure of whole rock and mineral δ^18^O values. MORB value is given as 5.7 ± 0.2‰ (ref. 64) and the sedimentary range is from 9 to 28.5‰ (refs 36, 65). Assimilation of crustal material (S-type granites, paragneisses or sediments) will increase δ^18^O values, whilst assimilation of high-temperature altered material will lower δ^18^O values. The Toba data are overall strongly displaced towards high-δ^18^O crustal values. (**b**) Binary mixing relationship between average YTT quartz core δ^18^O_magma_ (SIMS) value (n = 78) and a high silica hydrothermally-altered contaminant with δ^18^O = 0‰ (assumed value as a result of high-temperature alteration, cf. refs 7, 9 and 31). Approximately 25% mixing and assimilation with a low-δ^18^O component is required to bring the average core δ^18^O values (representing the ambient magma) to the lowest measured quartz rim value, reflecting a mixture of ambient magma and low-δ^18^O material for those portions of the YTT system that crystallised the low δ^18^O quartz rims. Binary mixing calculation of ambient YTT rhyolite with a basalt replenishment is provided in Supplementary Figure 1.

The δ^18^O_magma_ values of the YTT previously recorded in zircon, and now also in quartz, are consistent with an open and dynamic magma reservoir that received variable crustal and magmatic inputs during its evolution (Fig. 5a). To explore the early quartz crystallisation history at Toba, we modeled how much assimilation of granitic crust with an average δ^18^O value of 10‰ would be required to achieve our average magma value of 8.3‰, equivalent to the average measured quartz core value of 8.8‰. We employ the following mass-balance mixing model:


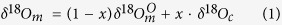


where 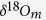 is the average quartz core δ^18^O_magma_ value (8.3‰), 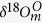 is the value of the starting magma, assumed here to have a δ^18^O_magma_ value of 7‰, 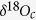 is the oxygen isotope ratio of the new component that enters the system, set here as 10‰ (i.e. a whole-rock value in equilibrium with our measured crustal quartz of 9.5‰), and *x* is the mass fraction of this component. Assuming a rhyolite magma with a δ^18^O starting value of 7‰ and a crustal contaminant with 10‰, then approximately 43% assimilation of high δ^18^O granitic crustal material would be required to produce the magma δ^18^O value in equilibrium with our average quartz core value. However, the exact amount of assimilation is difficult to constrain due to the wide variation in crustal δ^18^O values, and because it is likely that the highest quartz core values may represent xenocrystals of crustal origin, found at Toba and elsewhere in the Sunda arc (e.g. refs 24 and 39). Our new Sr-Nd-He isotopic data corroborate the oxygen isotope data and provide further evidence for addition of significant amounts of continental crust to the Toba magmatic system; however, it remains an open question whether this crustal component was added in the lower or upper crust or a combination of both. Irrespective of the exact origin of the high δ^18^O crustal component, the long-term assembly of Toba magma(s) via mantle and particularly crustal contributions would be expected to result in a diverse crystal cargo (cf. refs 38 and 40) and would be an effective way to explain the high δ^18^O and ^87^Sr/^86^Sr values and low ^143^Nd/^144^Nd and He ratios that characterise the Toba Tuffs (e.g., refs 24, 28, 38 and 41).

## Origin of the low δ^18^O component at Toba

If the YTT quartz dataset is considered as a whole, a t-test indicates that there is no significant difference between the cores (mean = 8.547; SD = 0.726) and rims (mean = 8.331; SD = 0.866); t (15) = 1.703, p = 0.109, but in addition to the commonly high δ^18^O values recorded in Toba quartz interiors, approximately 40% of the quartz crystals analysed by SIMS have lower δ^18^O values in their outer growth zones (Fig. 4a–f). A comparison of Figs 3 and 4 thus underlines that the Toba system demonstrates both large-scale homogenisation and crystal-scale heterogeneity characteristic of large silicic systems (cf. ref. 42). In order to evaluate rapid magmatic processes in large systems such as Toba, the key geochemical signals to evaluate are therefore subtle intra-crystal variations. As noted above, a percentage of 40% of crystals exist where downward changes in δ^18^O values are observed with progressive crystal growth (i.e. time), which thus requires the input of a much lower δ^18^O magma component than what would be inferred from bulk rock data and most bulk crystal analyses. Moreover, many cores and rims – apart from the outermost ones – probably grew at different times in different parts of the magma reservoir due to intra-reservoir convection and remobilisation processes^42^. The quartz crystals in the YTT have thus inherited a range of δ^18^O values, which when averaged produce a similar value overall. While we cannot always be sure that we have sampled the very outer rims of a crystal, the fact that intra-crystal changes tend to progress to lower values in some 40% of crystals analysed would imply that this incoming magma was recorded only in certain parts of the gigantic YTT magma reservoir, and thus effectively during the closing stages of pre-eruptive YTT chamber evolution.

Such a change towards relatively low δ^18^O values is also consistent with the concept of late-stage compositional diversification proposed previously for Toba^12^,^38^,^43^, and similar high-silica systems, such as Yellowstone or the Snake River Plain^7^,^9^. It is important to note that such a decrease in δ^18^O values cannot result from normal closed-system crystal fractionation. In addition, due to the preserved primary textures and intra-crystal compositional variations, we can exclude any post-crystallisation fluid overprint, which would result in irregular domains that reflect fluid fronts. Therefore, either basaltic replenishment with mantle-like δ^18^O values could have occurred^12^ or low-δ^18^O hydrothermally altered materials present in the shallow crust were added to the crystallising magma during the final stages of magmatic evolution, perhaps in a fashion similar to what was recently suggested for Yellowstone and for the Snake River Plain rhyolites^7^,^9^,^44^. In order to quantify the addition of a low-δ^18^O component to the YTT system, we again employ the mass-balance mixing model given in equation 1 above. In this model, 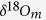 is the lowest recorded oxygen isotope value in the crystal outer zone (δ^18^O_magma_ value = 6.2‰), while 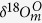 is the value of the starting magma, approximated here by the average quartz core δ^18^O_magma_ value (8.3‰). As before, 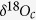 is the oxygen isotope ratio of the new component that enters the system, and *x* is the mass fraction of this component. To provide a first order assessment, we used a relatively normal mantle-type δ^18^O value of 5.5‰ for basalt replenishment^35^, and we consider hydrothermally-altered volcanic material from the caldera roof to have δ^18^O = 0‰, as has been suggested for comparable high silica systems elsewhere^7^,^44^.

Our modelling suggests that replenishment by basaltic magma with δ^18^O = 5.5‰ would be required in a proportion of ~75% basalt to 25% rhyolite (at δ^18^O = 8.3‰) to shift host magma to a δ^18^O_magma_ composition identical to values calculated from the lowest quartz rim values of our study (lowest quartz rim δ^18^O_magma_ = 6.2‰; Supplementary Figure 1). However, this proportion of mafic magma is unrealistic because quartz would no longer crystallise from a mixed magma of such an intermediate composition since the SiO_2_ content would be far too low to form quartz^45^. Indeed, a melt SiO_2_ content exceeding 70 wt.% is required to crystallise quartz at the pressure of the YTT magma chamber^23^,^45^. Furthermore, mafic replenishment would be expected to produce low-δ^18^O rims grown directly onto resorption surfaces due to contact with mafic magma^12^ and this is not observed. More generally, the role of mafic replenishment in driving super-eruptions is debated and indeed they may not affect super-eruption type magma reservoirs significantly because incremental mafic replenishments would not usually over-pressurise or pervasively alter the composition of such a large reservoir (cf. refs 46 and 47). However, mafic replenishments can act as a heat source if there is underplating or injection into the lowermost parts of a silicic magma reservoir^42^. Crystal zones that formed following underplating could reflect a magma hotter than recorded by previous rims. In other words, the parental magma batches would have been heated to temperatures above zircon and quartz saturation at a given δ^18^O value, such that this hotter magma would have promoted assimilation of roof material. Subsequent cooling below zircon and quartz saturation would crystallise these minerals with diverse δ^18^O values, which would explain, at least in part, the degree of the isotopic diversity observed in YTT zircon and quartz. High-temperature hydrothermally-altered roof and wall material surrounding the magma reservoir is an therefore alternative low-δ^18^O component that could have been added, especially given the observation that widespread low-δ^18^O crustal country rocks are probably not abundant beyond larger volcanic centres in this part of the globe^36^. Hydrothermally-altered roof rocks can have δ^18^O values of ~0‰ or lower due to prolonged high-temperature interaction with meteoric waters^7^,^9^,^44^,^48^. In the case of Toba, the reservoir roof and wall rocks are either composed of silica-rich magmatic rocks or pre-existing granitoids and are probably heavily fractured due to previous caldera collapses^15^. These rock types are fusible over short timescales, especially when hydrated and hydrothermally overprinted^43^,^48^,^49^,^50^. Our model suggests that admixing ~25% of a rhyolitic melt derived from a high-temperature altered roof rock with a δ^18^O value of 0‰ can reproduce the lowest δ^18^O rim values of the analysed YTT quartz (Fig. 5b). This amount of assimilation is energetically feasible and in line with estimates from other large caldera systems (e.g., ref. 44). For these reasons, we favour pre-eruptive addition of low-δ^18^O hydrothermally altered crustal melts to explain our new SIMS oxygen data.

A corollary of the roof assimilation model is the initiation of a positive feedback loop wherein explosive shattering of H_2_O-rich silicic rock on contact with magma could accelerate assimilation^51^. Moreover, after several cycles of caldera-forming activity at Toba, the reservoir roof (i.e. the caldera floor rocks) would be highly fractured and liable to mechanical break-up by piecemeal intra-caldera structures on a variety of scales (e.g., refs 15 and 52). Such a faulted and fractured roof would provide pathways for infiltrating hydrothermal fluids, and would provide an easily mobilised reservoir of low-δ^18^O material spatially adjacent to the magma reservoir. Indeed, mechanical roof failure and roof subsidence into the magma reservoir is a highly probable eruption trigger at large caldera systems such as Toba^52^,^53^, especially as this caldera sits upon the Batak Tumor, an enormous swell or tumescence area around Lake Toba, which is related to emplacement of the large Toba reservoir and probably also to buoyant magma rise^15^ (Fig. 1). Associated roof uplift from emplacement and rise of silicic magma within this swell will have imparted an extensional regime onto the reservoir roof (e.g., refs 15, 46, 47 and 52), which would thus have been intensely fractured and weakened already prior to the YTT event and hence made susceptible to partial collapse and rapid recycling during caldera unrest.

## Linking assimilation with timescales

Seven of the 15 YTT quartz crystals examined here for their δ^18^O values were previously analysed for Ti-in-quartz diffusion to derive residence timescales^12^. Ti-in-quartz showed that the outer YTT quartz rims on the studied crystals had an average residence time on the order of 10^2^ years, which most likely reflects the period of final crystal growth prior to eruption. On the other hand, chemical diversification already commenced some 35 ka before the final YTT event^38^, and likely involved mafic underplating and small to medium-sized replenishments (cf. ref. 54). However, our new δ^18^O data lead us to suggest that chemical diversification of the YTT magma may have been influenced by crustal assimilation and progressively more pronounced self-assimilation that recycled part of the volcanic superstructure due to increasing roof instability in the run-up to the cataclysmic YTT event. Numerical experiments have already highlighted that hydrated volcanic material can melt over very short timescales even in large silicic caldera settings and at rates of many metres per year^43^. Such timescales are in agreement with those established for the YTT system^12^,^38^.

**Figure 6:**
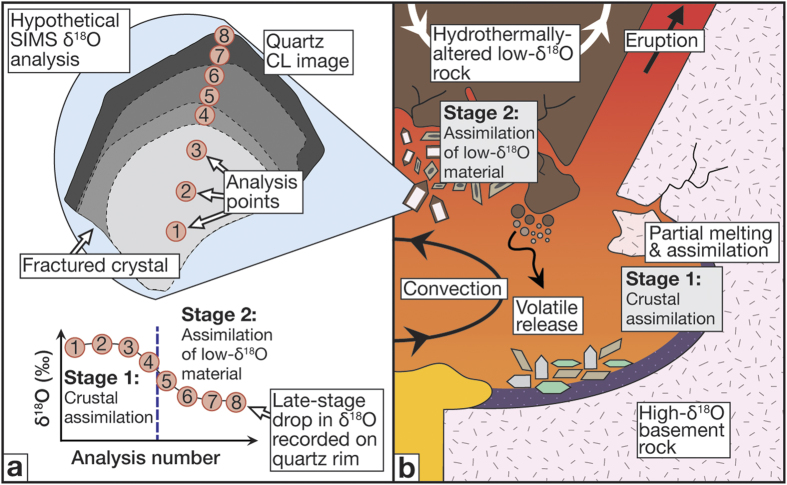
Conceptual sketch of quartz isotope stratigraphy and magma reservoir dynamics. (**a**) The variations recorded in quartz crystal chemistry demonstrate the utility of crystal oxygen isotope stratigraphy to fingerprint silicic magma evolution at high spatial resolution. (**b**) The results of this study are interpreted to reflect uptake of a high δ^18^O component from the crust before quartz saturation and consequent crystallisation (Stage 1), and subsequent assimilation of low-δ^18^O roof materials by the YTT magma (Stage 2) during the final period of caldera unrest prior to the cataclysmic YTT eruption. This stage may have lasted several hundred years. Note that the timing of Stage 1 is less certain, and if the highest δ^18^O quartz cores represent xenocrystals derived from the crust then the high δ^18^O component could have been introduced tens of thousands of years before the YTT eruption (see text for details).

Late-stage addition of high-silica, hydrated, and hence fertile crustal material from the reservoir roof, as supported by our δ^18^O quartz data, would likely supply extra volatiles to the resident magma and so could promote build-up of sufficient volatile over-pressure to initiate an eruption (e.g., refs 55, 56, 57; Supplementary Figure 2). The source of these extra volatiles could be (i) water-filled vesicles in assimilated roof material (e.g. ref. 58), or (ii) assimilation of hydrothermally altered clay-rich intra-caldera materials with high H_2_O contents (e.g. >6 wt.%, ref. 59). Furthermore, stoping may have been an important mechanism to swiftly introduce hydrous roof material, leading to initial degassing from clays and volatile-filled vesicles of the stoped material, followed by partial hydrous melting, and, if left for sufficient time at high temperature, bulk melting of the solid residue of the stoped materials. Due to its high silica nature, and upper crustal storage level, we envisage that the Toba system would have rapidly achieved volatile oversaturation upon assimilation of hydrous roof material, which may explain why only 40% of our quartz crystals record a late-stage drop in their δ^18^O values (e.g., Fig. 6). A large portion of the resident magma (>60%) likely erupted before its δ^18^O value was significantly lowered by the late-stage low-δ^18^O assimilant (cf. refs 43 and 57).

The wider implications of our results are that the large size of quartz crystals and its high oxygen diffusion rates at magmatic temperatures (10^4^ times faster than zircon^10^,^11^) now allow us to resolve events on the order of hundreds of years prior to eruption and to assess the degree of late-stage crustal assimilation. As illustrated in Fig. 6, the spatial resolution of SIMS analysis of quartz can provide a level of detail that complements whole-rock and zircon studies (cf. refs 9 and 38), and, in the case of Toba, records an otherwise undetected change in the δ^18^O values of the outer zones of a portion of the studied quartz crystals.

## Methods Summary

The YTT whole rock and pumice samples were crushed, sieved, cleaned and picked for pristine quartz, feldspar, biotite and amphibole. For CL characterisation, quartz grains were mounted in epoxy resin and analysed with a CL detector attached to a JEOL microprobe at the Department of Earth Sciences at Bristol University. Whole rock powders were analysed for oxygen isotopes using a conventional line and mineral grains by using a laser fluorination line at the University of Cape Town. For SIMS analysis, quartz crystals were fixed in epoxy mounts and coated with gold. *In-situ* O-isotope measurements were made using a CAMECA IMS 1280 SIMS instrument at the Swedish Museum of Natural History, Stockholm (NordSIM). A 20 keV Cs^+^ primary beam of ca. 2.5 nA was used in critically-focussed mode together with a 5 μm raster to sputter a ca. 10 μm sample area. The runs comprised a 90 second pre-sputter period with a raster of 20 μm, and field aperture centering using the ^16^O signal followed by 64 seconds of data acquisition using two Faraday detectors in the multicollector system operating a common mass resolution of ca. 2500. Corrections for instrumental mass fractionation were determined using international reference material NBS-28 (silica sand), with further details given in refs 60 and 61. Full descriptions of all methods employed, including for Sr-, Nd-, and He-isotope analyses, are provided in the Supplementary Information.

## Additional Information

**How to cite this article**: Budd, D. A. *et al*. Magma reservoir dynamics at Toba caldera, Indonesia, recorded by oxygen isotope zoning in quartz. *Sci. Rep.* **7**, 40624; doi: 10.1038/srep40624 (2017).

**Publisher's note:** Springer Nature remains neutral with regard to jurisdictional claims in published maps and institutional affiliations.

## Supplementary Material

Supplementary Information
