# Supplementary material for: Magma reservoir dynamics at Toba caldera, Indonesia, recorded by oxygen isotope zoning in quartz
Source: Sci Rep. 2017 Jan 25;7:40624. doi: 10.1038/srep40624 (PMC5264179; doi:10.1038/srep40624)
Supplement: Supplementary Information [file srep40624-s1.pdf]

Magma reservoir dynamics at Toba caldera, Indonesia, recorded by oxygen isotope zoning in quartz

David A. Budd, Valentin R. Troll, Frances M. Deegan, Ester M. Jolis, Victoria C. Smith, Martin J. Whitehouse, Chris Harris, Carmela Freda, David R. Hilton, Saemundur A. Halldórsson, Ilya N. Bindeman

Supplementary Information

Contents:

- **Methods**
- **Supplementary discussion**
- **Supplementary Figure 1:** Mass balance modeling of mafic replenishment at Toba.
- **Supplementary Figure 2:** Mass balance modeling of assimilation of hydrated material at Toba.
- **Supplementary Table 1.** Whole rock geochemical data (major element oxides) from Toba and surrounding basement obtained by ICP-ES.
- **Supplementary Table 2.** All $\delta^{18}O$ values obtained for whole rocks (conventional fluorination) and single minerals (laser fluorination). All values reported in δ-notation and expressed in per mil (‰) relative to SMOW (1σ error = 0.15‰). Also shown are $^{87}Sr/^{86}Sr$ and $^{143}Nd/^{144}Nd$ isotopic data (1σ error = 0.000013) and $^{3}He/^{4}He$ (R_A) data (1σ error = 0.055).
- **Supplementary Table 3.** All $\delta^{18}O$ values from quartz SIMS analysis. All values reported in δ-notation and expressed in per mil (‰) relative to SMOW.

Methods

Sample selection: The YTT was sampled from tuff units exposed along the caldera walls surrounding the Toba caldera. Whole rock and pumice samples employed in this study were free from alteration and uniform in colour (documented in **Supplementary Table 1)**. In thin section, all samples have a similar mineral assemblage, comprising mainly quartz, feldspar, biotite and amphibole, with up to 40 % crystal content. Bulk analyses of rocks and pumices reveal SiO_2 contents between 71-73 wt.%, consistent with the range of pumice chemistries identified (68-77 wt.% SiO_2; ref 1). A portion of the quartz crystals analysed in this study were previously analysed by[2] and were extracted from pumice fragments with ~75 wt.% SiO_2. They are hence extensively documented and well characterised in the referenced study.

Bulk rock elemental analyses: Bulk rock major element oxides were analysed using ICP-ES at Acme Labs, Vancouver, Canada, following lithium metaborate/tetraborate fusion and dilute nitric digestion **(Supplementary Table 1)**. Trace elements were analysed by ICP-MS after preparation by multi-acid digestion. Loss-on-ignition, and hence volatile content, was determined by weight difference after ignition at 1000 °C. Reproducibility was monitored by repeat measurements of internal standards (Acme Labs; www.acmelab.com) and are available upon request.

He isotope analyses: For He isotope analysis, fresh pyroxene crystals were picked under a binocular microscope from crushed whole rock material from the HDT. We note that the YTT is virtually devoid of pyroxene and hence it was unfeasible to extract sufficient crystals for helium isotope analysis. Helium isotopes were measured using a MAP215 noble gas mass spectrometer at the Fluids and Volatiles Laboratory, Scripps Institution of Oceanography, University of California San Diego. La Jolla air He (=1 R_A) prepared and run under identical experimental conditions to samples, was used as a standard, following the methods described in ref. 3.

Sr-Nd isotope analyses: The strontium isotope data were obtained at the Vrije Universiteit Amsterdam (VUA) using a Finnigan MAT 262 TIMS system operating in static mode. Sample treatment and analytical details are given in[4], and references therein. Neodymium isotopes were also analysed at the VUA using a Finnigan Neptune Multi Collector Inductively Coupled Mass Spectrometer (MC-ICP-MS) following the method described in[5]. Full details of the method

used, the instrument settings, analytical reproducibility and data reduction are given in[6]. The MC-ICP-MS and TIMS data quality were monitored by repeated analysis of both internal lab standards and international reference materials (BHVO-2) as reported in[7]. Blanks run during the course of analysis were negligible[7].

CL characterisation: Quartz crystals were imaged using a CL detector mounted on a JEOL electron microprobe in the Department of Earth Sciences at the University of Bristol, using the same methods as[2]. Beam conditions were 10 kV and 40 nA current, with a pixel resolution of 1000 x 750, where 1 pixel is 3.5 µm.

Whole rock and mineral oxygen isotope analyses: Whole rock and mineral grain oxygen isotope analyses of quartz, feldspar, biotite and amphibole were carried out at the University of Cape Town, South Africa, using a conventional line and a laser fluorination line, respectively **(Supplementary Table 2)**. The results are reported in standard δ-notation relative to SMOW (Standard Mean Ocean Water), using the internal NBS-28 quartz standard ($\delta^{18}O$ = 9.64 ‰) to normalise the raw data. All values are consistent with O-isotope equilibrium at magmatic temperatures. In practice, the difference between the raw and normalised data was less than ca. 0.5 ‰. During the course of this study, the analytical error based on multiple analyses of the standard was ca. ± 0.15 ‰. Full analytical details of the laser line are given in[9]. Measured values of the UCT internal standard MON GT[10] were used to normalise the raw data and correct for drift in the reference gas. The average difference in $\delta^{18}O$ values of duplicates of MON GT analysed during this study was 0.14 ‰, and corresponds to a 2σ value of 0.19 ‰.

In-situ SIMS oxygen isotope analyses: Following CL and EPMA characterisation, selected pristine quartz crystals were analysed by SIMS at the Nordsim ion microprobe facility at the Swedish Museum of Natural History, Stockholm, using a CAMECA IMS 1280 instrument. Quartz crystals were mounted in epoxy resin, polished, and coated in gold prior to $\delta^{18}O$ analysis along core-rim transects during two separate analytical sessions **(Supplementary Table 3)**. SIMS instrumentation and methods are based on[11,12] with modifications. A 20 keV Cs^{+} primary beam of ca. 2.5 nA was used in critically-focussed mode together with a 5µm raster to sputter a ca. 10 µm sample area. A normal incidence low energy electron gun provided charge compensation. The runs comprised a 90 second pre-sputter period with a raster of 20 µm, and field aperture centring using ^{16}O signal followed by 64 seconds of data acquisition using two Faraday detectors in the multicollector system that operates at a common mass resolution of ca.

2500. The secondary magnet field was regulated at high precision using a Metrolab NMR teslameter. Instrumental mass fractionation was determined using as reference NBS-28 (silica sand) with a $\delta^{18}O_{NBS}$ value of +9.50‰ (ref 13) and ratios were corrected accordingly. Two analyses of the reference material were run between every four unknown analyses. Internal precision (1σ mean) on $\delta^{18}O$ for both the NBS-28 reference material and the unknowns ranged from ±0.41 ‰ to ±0.52 ‰ for the first session and ±0.33 ‰ to ±0.43 ‰ for the second session. The external error on the NBS-28 ranged from ±0.32 ‰ to 0.40 ‰ (RSD; n=106). External errors were propagated into the overall analytical error for each analysis.

References for methods

1. Chesner, C. Petrogenesis of the Toba Tuffs, Sumatra, Indonesia. *J. Petrol.* **39,** 397–438 (1998).
2. Matthews, N. E., Huber, C., Pyle, D. M. & Smith, V. C. Timescales of Magma Recharge and Reactivation of Large Silicic Systems from Ti Diffusion in Quartz. *J. Petrol.* **53,** 1385–1416 (2012).
3. Hilton, D. R. *et al.* Helium isotopes at Rungwe Volcanic Province, Tanzania, and the origin of East African Plateaux. *Geophys. Res. Lett.* **38,** (2011).
4. Deegan, F. M. *et al.* Crustal versus source processes recorded in dykes from the Northeast volcanic rift zone of Tenerife, Canary Islands. *Chem. Geol.* **334,** 324–344 (2012).
5. Luais, B., Telouk, P. & Albarede, F. Precise and accurate neodymium isotopic measurements by plasma-source mass spectrometry. *Geochim. Cosmochim. Acta* **61,** 4847–4854 (1997).
6. Elburg, M., Vroon, P., Vanderwagt, B. & Tchalikian, A. Sr and Pb isotopic composition of five USGS glasses (BHVO-2G, BIR-1G, BCR-2G, TB-1G, NKT-1G). *Chem. Geol.* **223,** 196–207 (2005).
7. Jolis, E. M. Magma-crust interaction at subduction zone volcanoes, PhD thesis, Uppsala University (2013).
8. Sandrin, A., Edfelt, Å., Waight, T. E., Berggren, R. & Elming, S.-Å. Physical properties and petrologic description of rock samples from an IOCG mineralized area in the northern Fennoscandian Shield, Sweden. *J. Geochemical Explor.* **103,** 80–96 (2009).

9. Vennemann, T. W. & Smith, H. S. The rate and temperature of reaction of ClF_3 with silicate minerals, and their relevance to oxygen isotope analysis. *Chem. Geol.* **86,** 83–88 (1990).
10. Harris, C., Smith, H. S. & le Roex, A. P. Oxygen isotope composition of phenocrysts from Tristan da Cunha and Gough Island lavas: variation with fractional crystallization and evidence for assimilation. *Contrib. to Mineral. Petrol.* **138,** 164–175 (2000).
11. Nemchin, A. A., Pidgeon, R. & Whitehouse, M. J. Re-evaluation of the origin and evolution of >4.2 Ga zircons from the Jack Hills metasedimentary rocks. *Earth Planet. Sci. Lett.* **244,** 218–233 (2006).
12. Whitehouse, M. J. & Nemchin, A. A. High precision, high accuracy measurement of oxygen isotopes in a large lunar zircon by SIMS. *Chem. Geol.* **261,** 32–42 (2009).
13. Gonfiantini, R., Stichler, W. & Rozanski, K. Standards and intercomparison materials distributed by the International Atomic Energy Agency for stable isotope measurements. *Int. At. Energy Agency stable Isot. Meas.* **13,** 1–18 (1995).

Supplementary discussion

Assimilation of hydrated material: We have modelled the release of volatiles (H_2O) from hydrated country rock upon assimilation into the YTT magma, which can affect the total H_2O content of the magma according to the following mass balance expression:

$$\boldsymbol{C_i = M_1 \times \alpha_i + M_2 \times \alpha_j}$$

$C_i = concentration\ (\%)\ of\ element\ \alpha_{i,j} in\ magma$

$M_{1,2}\ =\ magma\ and\ crustal\ volume\ respectively$

$\alpha_{i,j} = fraction\ of\ component$

Our model varies the degree of assimilation from 5 to 25 % and the H_2O contents in the hydrated country rock from 4 to 12 %. The starting value of 4% H_2O corresponds to the lowest measured value from a quartz melt inclusion study of the YTT[1]. The model predicts that 25 % assimilation of a country rock with 10 % H_2O raises the H_2O content of a resident magma by 1.3 times **(Supplementary Figure 2)**. Previous work suggests that the extra volume change caused by the addition of even relatively minor amounts of volatiles can create overpressures sufficient to overcome the tensile strength of country rock and this can initiate an eruption (cf. ref 2). The model results support our hypothesis that the incorporation of hydrated reservoir roof rock can prime the caldera system for eruption. Mafic injections, in turn, likely have lower volatile contents than hydrated roof and country rock, yet they supply new heat into the system to sustain the reservoir and facilitate large-scale melting of the hydrothermally-altered reservoir roof rocks.

References for supplementary discussion

1. Chesner, C. A. & Luhr, J. F. A melt inclusion study of the Toba Tuffs, Sumatra, Indonesia. *J. Volcanol. Geotherm. Res.* **197,** 259–278 (2010).
2. Tait, S., Jaupart, C. & Vergniolle, S. Pressure, gas content and eruption periodicity of a shallow, crystallising magma chamber. *Earth Planet. Sci. Lett.* **92,** 107–123 (1989).

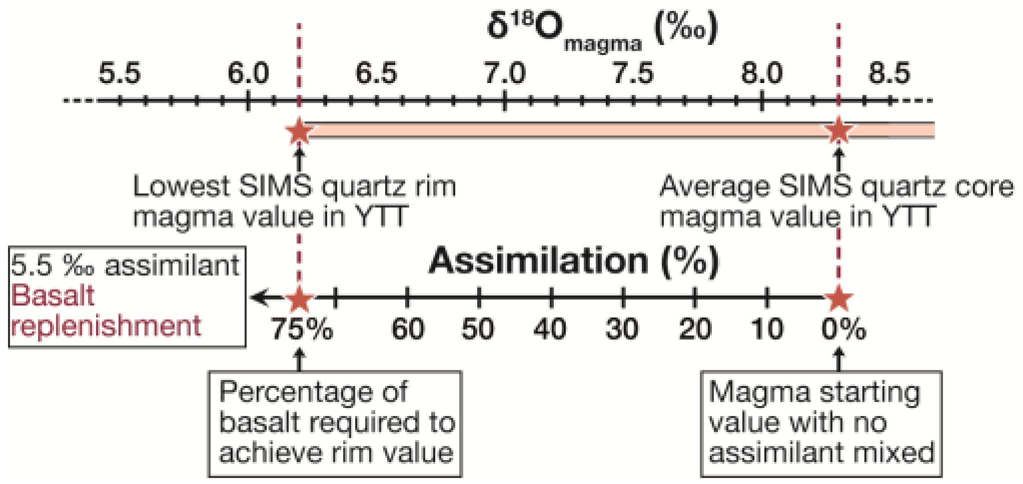

Supplementary Figure 1: The effect of a primitive basalt replenishment ($\delta^{18}O$ = 5.5 ‰) on the average quartz core $\delta^{18}O$ (SIMS) value requires input of ~75 % of basalt to the Toba rhyolite magma that crystallised the high $\delta^{18}O$ quartz cores to bring the $\delta^{18}O$ value down to the lowest measured quartz rim $\delta^{18}O_{magma}$ value (6.2 ‰). Such an admixture would not allow for continued quartz growth as it would result in an intermediate composition (i.e., an andesite magma) that would no longer crystallise quartz.

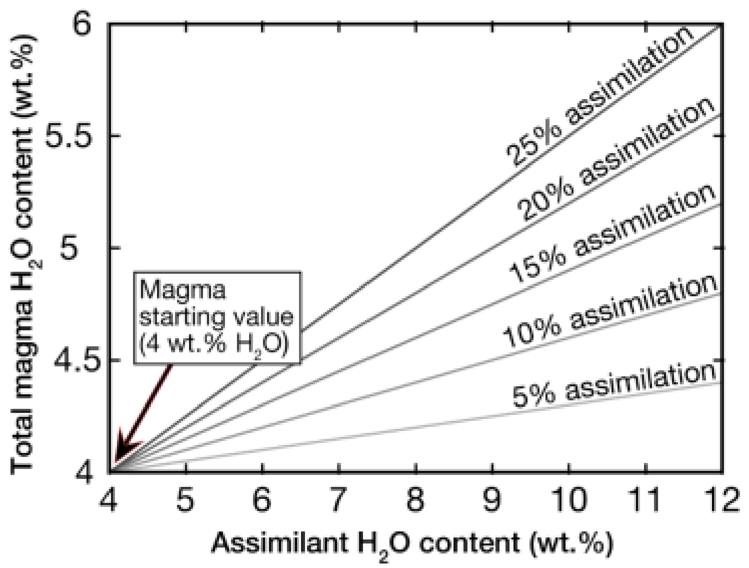

Supplementary Figure 2: Plot of crustal H_2O contribution to a rhyolite magma system at different degrees of assimilation. Hypothetical initial magma H_2O content is set at 4 wt. % (ref. 66) and H_2O content of the assimilant is varied between 4 and 12 wt. %. Assuming assimilation of a rock with 10 wt. % H_2O, an increase in magma H_2O by a factor of 1.4 is calculated, which may provide the extra volatile pressure to a system already in a state of critical unrest.

Table S1. Whole rock geochemical data (major element oxides) from Toba and surrounding basement obtained by ICP-ES.

Sample Name	Rock type	SiO_2	TiO_2	Al_2O_3	Fe_2O_3	MnO	MgO	CaO	Na_2O	K_2O	LOI	Total
SU-YTT-D-56A	Rhyolite tuff	70.81	0.18	14.68	1.44	0.05	0.36	1.75	3.34	4.62	2.6	99.83
SU-YTT-D-56B	Rhyolite tuff	70.77	0.19	14.69	1.60	0.05	0.37	1.88	3.39	4.6	2.3	99.84
SU-YTT-D-56C	Rhyolite tuff	70.73	0.19	14.90	1.39	0.05	0.36	1.85	3.35	4.64	2.4	99.86
SU-YTT-D-57A	Rhyolite tuff	70.80	0.18	14.47	1.88	0.07	0.38	1.93	3.47	4.51	2.2	99.89
SU-YTT-D-57B	Rhyolite tuff	70.74	0.18	14.40	1.85	0.07	0.36	1.92	3.46	4.56	2.3	99.84
SU-YTT-D-58	Rhyolite tuff	70.59	0.18	14.56	1.38	0.05	0.34	1.82	3.17	4.77	3.0	99.86
SU-YTT-PUM-66	Pumice	72.72	0.16	13.13	1.63	0.07	0.32	1.19	2.68	5.19	2.8	99.89
SU-HDT-83	Dacite tuff	61.66	0.69	15.78	6.37	0.11	3.02	5.51	2.48	2.99	1.1	99.71
SU-HDT-86	Dacite tuff	61.53	0.68	15.94	6.26	0.10	2.77	5.43	2.52	3.15	1.3	99.68
SU-BS	Granitoid basement	75.18	0.14	12.83	1.65	0.05	0.27	0.89	3.14	5.08	0.7	99.93
SU-YTT-BN3	Granitoid basement	67.05	0.36	15.57	2.69	0.04	1.72	3.37	4.42	2.78	1.7	99.71

Table S2. All δ^{18}O values obtained for whole rocks (conventional fluorination) and single minerals (laser fluorination). All values reported in δ-notation and expressed in per mil (‰) relative to SMOW (1σ error = 0.15‰). Also shown are $^{87}Sr/^{86}Sr$ and $^{143}Nd/^{144}Nd$ isotopic data (1σ error = 0.000013) and $^{3}He/^{4}He$ (R_A) data (1σ error = 0.055).

Sample name	Sequence	Whole rock δ^{18}O (‰)	Quartz δ^{18}O (‰)	Feldspar δ^{18}O (‰)	Biotite δ^{18}O (‰)	Amphibole δ^{18}O (‰)	$^{87}Sr/^{86}Sr$	$^{143}Nd/^{144}Nd$	Helium ($^{3}He/^{4}He$)
SU-YTT-D-56A	YTT		8.4		7.1				
SU-YTT-D-56B	YTT	9.8	9.3		7.3				
SU-YTT-D-56C	YTT	9.9	9.0		7.1				
SU-YTT-D-57A	YTT	9.4	8.9		6.8				
SU-YTT-D-57B	YTT	9.3	8.9		6.8				
SU-YTT-D-58	YTT	9.0	9.0		8.9				
SU-YTT-D-62A	YTT	8.5	8.2, 8.9	7.7, 7.7	6.8	5.5, 6.2	0.714033 (±13)	0.512113 (±13)	
SU-YTT-D-62B	YTT	9.5	9.1	7.9					
SU-YTT-D-62C	YTT	8.7	8.9		6.8	6.4			
SU-YTT-D-70	YTT	8.5	8.9	7.6		5.5			
SU-YTT-D-71	YTT	8.5	9.3	8.0					
SU-YTT-D-72	YTT	8.2	8.9	8.1					
SU-HDT-83	HDT	8.6							1.76, 0.67
SU-BS	Basement granitoid		9.5						
SU-YTT-BN3	Basement granitoid		9.4						
SU-BS4	Basement granitoid		6.7, 6.4, 6.5						

Table S3. All δ^{18}O values from quartz SIMS analysis. All values reported in δ-notation and expressed in per mil (‰) relative to SMOW.

Sample name	Analysis point	Rim(R)/ Core(C)	Drift corrected data $^{18}O/^{16}O$		$\delta^{18}O$ quartz (‰)	Error (±1σ)
SU-YTT-C1	1	C	0.00201154	± 38	8.75	0.44
SU-YTT-C1	2	C	0.00201191	± 30	8.94	0.42
SU-YTT-C1	3	C	0.00201103	± 44	8.50	0.45
SU-YTT-C1	4	C	0.00200995	± 34	7.96	0.43
SU-YTT-C1	5	C	0.00201095	± 52	8.46	0.47
SU-YTT-C1	6	C	0.00201057	± 33	8.27	0.43
SU-YTT-C1	7	R	0.00201023	± 38	8.10	0.44
SU-YTT-C1	8	R	0.00200951	± 47	7.74	0.46
SU-YTT-C2	1	R	0.00200937	± 39	7.66	0.44
SU-YTT-C2	2	R	0.00201028	± 29	8.12	0.42
SU-YTT-C2	3	R	0.00201125	± 27	8.61	0.42
SU-YTT-C2	4	R	0.00201080	± 46	8.38	0.45
SU-YTT-C2	5	R	0.00201116	± 43	8.56	0.45
SU-YTT-C2	6	R	0.00201109	± 36	8.53	0.43
SU-YTT-C2	7	R	0.00200967	± 40	7.82	0.44
SU-YTT-C2	8	R	0.00201169	± 41	8.83	0.45
SU-YTT-C2	9	R	0.00200945	± 28	7.71	0.42
SU-YTT-C2	10	C	0.00200989	± 41	7.93	0.44
SU-YTT-C3	1	C	0.00201258	± 35	9.28	0.43
SU-YTT-C3	2	C	0.00201148	± 17	8.73	0.40
SU-YTT-C3	3	R	0.00200974	± 33	7.85	0.43
SU-YTT-C3	4	R	0.00200899	± 31	7.48	0.42
SU-YTT-C3	5	R	0.00200942	± 37	7.69	0.43
SU-YTT-C4	1	R	0.00201007	± 46	8.02	0.46
SU-YTT-C4	2	R	0.00201027	± 40	8.12	0.44
SU-YTT-C4	3	C	0.00200990	± 42	7.93	0.45
SU-YTT-C4	4	C	0.00201019	± 30	8.08	0.42
SU-YTT-C5	1	C	0.00201009	± 43	8.03	0.45
SU-YTT-C5	2	C	0.00201047	± 31	8.22	0.42
SU-YTT-C5	3	C	0.00200933	± 37	7.64	0.44
SU-YTT-C5	4	C	0.00200913	± 31	7.55	0.42
SU-YTT-C5	5	R	0.00200924	± 57	7.60	0.49
SU-YTT-C5	6	R	0.00200949	± 49	7.73	0.46
SU-YTT-C5	7	R	0.00200885	± 48	7.41	0.46
SU-YTT-C5	8	R	0.00200861	± 44	7.28	0.45
SU-YTT-C5	9	R	0.00200871	± 56	7.34	0.48
SU-YTT-C6	1	C	0.00200934	± 47	7.65	0.46
SU-YTT-C6	2	C	0.00200798	± 60	6.97	0.49
SU-YTT-C6	3	R	0.00200900	± 51	7.48	0.47
SU-YTT-C6	4	R	0.00200919	± 33	7.58	0.43
SU-YTT-C6	5	R	0.00200913	± 44	7.55	0.45
SU-YTT-C6	6	R	0.00200762	± 36	6.79	0.43

Table S3. Continued.

Sample name	Analysis point	Rim(R)/ Core(C)	Drift corrected data $^{18}O/^{16}O$		$\delta^{18}O$ quartz (‰)	Error (±1σ)
SU-YTT-C6	7	R	0.00200803	± 40	6.99	0.44
SU-YTT-C6	8	R	0.00200746	± 34	6.71	0.43
SU-YTT-C6	9	R	0.00200762	± 34	6.79	0.43
SU-YTT-C7	1	C	0.00200949	± 31	7.73	0.42
SU-YTT-C7	2	C	0.00200868	± 34	7.32	0.43
SU-YTT-C7	3	C	0.00200916	± 53	7.56	0.47
SU-YTT-C7	4	C	0.00200981	± 49	7.89	0.46
SU-YTT-C7	5	C	0.00200852	± 34	7.24	0.43
SU-YTT-C7	6	C	0.00200880	± 42	7.38	0.45
SU-YTT-C7	7	C	0.00200821	± 59	7.08	0.49
SU-YTT-C7	8	C	0.00200954	± 35	7.75	0.43
SU-YTT-C7	9	R	0.00200839	± 42	7.17	0.45
SU-YTT-C8	1	C	0.00201048	± 38	8.22	0.44
SU-YTT-C8	2	C	0.00201118	± 50	8.57	0.47
SU-YTT-C8	3	R	0.00201034	± 50	8.15	0.47
SU-YTT-C8	4	R	0.00200960	± 43	7.78	0.45
SU-YTT-C8	5	R	0.00200915	± 39	7.56	0.44
SU-YTT-C8	6	R	0.00200865	± 40	7.31	0.44
SU-YTT-C8	7	R	0.00200881	± 56	7.39	0.48
SU-YTT-C9	1	R	0.00201834	± 37	9.93	0.37
SU-YTT-C9	2	R	0.00201795	± 28	9.73	0.35
SU-YTT-C9	3	R	0.00201772	± 22	9.61	0.34
SU-YTT-C9	4	R	0.00201743	± 21	9.47	0.33
SU-YTT-C9	5	R	0.00201787	± 21	9.69	0.34
SU-YTT-C9	6	R	0.00201872	± 27	10.12	0.34
SU-YTT-C9	7	R	0.00201771	± 25	9.61	0.34
SU-YTT-C9	8	R	0.00201836	± 26	9.93	0.34
SU-YTT-C9	9	R	0.00201849	± 31	10.00	0.35
SU-YTT-C9	10	R	0.00201711	± 33	9.31	0.36
SU-YTT-C9	11	R	0.00201999	± 35	9.81	0.37
SU-YTT-C9	12	R	0.00201910	± 22	9.37	0.35
SU-YTT-C9	13	R	0.00201974	± 18	9.69	0.34
SU-YTT-C9	14	R	0.00201992	± 42	9.78	0.39
SU-YTT-C9	15	R	0.00201953	± 24	9.58	0.35
SU-YTT-C9	16	R	0.00201945	± 32	9.54	0.36
SU-YTT-C9	17	R	0.00202015	± 31	9.89	0.36
SU-YTT-C9	18	R	0.00201921	± 22	9.42	0.34
SU-YTT-C9	19	R	0.00201898	± 24	9.31	0.35
SU-YTT-C9	20	R	0.00201883	± 26	9.23	0.35
SU-YTT-C9	21	R	0.00202053	± 29	10.08	0.36
SU-YTT-C9	22	R	0.00201937	± 37	9.50	0.38
SU-YTT-C9	23	R	0.00201838	± 33	9.01	0.37
SU-YTT-C9	24	R	0.00201780	± 26	9.66	0.34

Table S3. Continued.

Sample name	Analysis point	Rim(R)/ Core(C)	Drift corrected data $^{18}O/^{16}O$		$\delta^{18}O$ quartz (‰)	Error (±1σ)
SU-YTT-C9	25	R	0.00201783	± 28	9.67	0.35
SU-YTT-C9	26	C	0.00201754	± 33	9.52	0.36
SU-YTT-C9	27	C	0.00201730	± 26	9.40	0.34
SU-YTT-C9	28	C	0.00201843	± 28	9.97	0.35
SU-YTT-C9	29	C	0.00201780	± 27	9.66	0.34
SU-YTT-C9	30	C	0.00201759	± 28	9.55	0.35
SU-YTT-C9	31	C	0.00201797	± 21	9.74	0.33
SU-YTT-C9	32	C	0.00201707	± 24	9.29	0.34
SU-YTT-C9	33	C	0.00201734	± 31	9.43	0.35
SU-YTT-C10	1	C	0.00201961	± 24	9.62	0.35
SU-YTT-C10	2	C	0.00202024	± 32	9.94	0.36
SU-YTT-C10	3	C	0.00201888	± 32	9.26	0.36
SU-YTT-C10	4	C	0.00201898	± 33	9.30	0.37
SU-YTT-C10	5	C	0.00201902	± 37	9.32	0.38
SU-YTT-C10	6	C	0.00201944	± 37	9.54	0.38
SU-YTT-C10	7	C	0.00201932	± 23	9.48	0.35
SU-YTT-C10	8	C	0.00202068	± 24	10.16	0.35
SU-YTT-C10	9	C	0.00202033	± 22	9.98	0.35
SU-YTT-C10	10	R	0.00201868	± 25	9.15	0.35
SU-YTT-C10	11	R	0.00201858	± 42	9.11	0.39
SU-YTT-C10	12	R	0.00201957	± 31	9.60	0.36
SU-YTT-C10	13	R	0.00201793	± 32	8.78	0.36
SU-YTT-C10	14	R	0.00201811	± 39	8.87	0.38
SU-YTT-C10	15	R	0.00201832	± 51	8.98	0.41
SU-YTT-C10	16	R	0.00201846	± 33	9.05	0.37
SU-YTT-C10	17	R	0.00201874	± 37	9.18	0.38
SU-YTT-C11	1	R	0.00201917	± 25	9.40	0.35
SU-YTT-C11	2	R	0.00201930	± 30	9.47	0.36
SU-YTT-C11	3	R	0.00201828	± 39	8.95	0.38
SU-YTT-C11	4	R	0.00201812	± 43	8.88	0.39
SU-YTT-C11	5	R	0.00201868	± 28	9.15	0.36
SU-YTT-C11	6	R	0.00201832	± 31	8.97	0.36
SU-YTT-C11	7	R	0.00201945	± 41	9.54	0.39
SU-YTT-C11	8	R	0.00201846	± 39	9.05	0.38
SU-YTT-C11	9	R	0.00201786	± 42	8.75	0.39
SU-YTT-C11	10	C	0.00201928	± 44	9.46	0.39
SU-YTT-C11	11	C	0.00201858	± 39	9.10	0.38
SU-YTT-C11	12	C	0.00201848	± 30	9.06	0.36

Table S3. Continued.

Sample name	Analysis point	Rim(R)/ Core(C)	Drift corrected data $^{18}O/^{16}O$		$\delta^{18}O$ quartz (‰)	Error (±1σ)
SU-YTT-C11	13	C	0.00201765	± 26	8.64	0.35
SU-YTT-C11	14	C	0.00201891	± 31	9.27	0.36
SU-YTT-C11	15	C	0.00201847	± 31	9.05	0.36
SU-YTT-C11	16	C	0.00201809	± 37	8.86	0.37
SU-YTT-C11	17	C	0.00201862	± 47	9.13	0.40
SU-YTT-C11	18	C	0.00201814	± 33	8.89	0.37
SU-YTT-C12	1	C	0.00201762	± 39	8.62	0.38
SU-YTT-C12	2	C	0.00201766	± 40	8.64	0.38
SU-YTT-C12	3	R	0.00201756	± 34	8.60	0.37
SU-YTT-C12	4	R	0.00201825	± 32	8.94	0.36
SU-YTT-C12	5	R	0.00201855	± 32	9.09	0.36
SU-YTT-C12	6	R	0.00201872	± 30	9.18	0.36
SU-YTT-C12	7	R	0.00201895	± 38	9.29	0.38
SU-YTT-C12	8	R	0.00201737	± 34	8.50	0.37
SU-YTT-C12	9	R	0.00201761	± 30	8.62	0.36
SU-YTT-C13	1	R	0.00201927	± 35	9.45	0.37
SU-YTT-C13	2	R	0.00201849	± 36	9.06	0.37
SU-YTT-C13	3	R	0.00201761	± 19	8.62	0.34
SU-YTT-C13	4	R	0.00201826	± 28	8.94	0.36
SU-YTT-C13	5	R	0.00201903	± 39	9.33	0.38
SU-YTT-C13	6	R	0.00201815	± 23	8.89	0.35
SU-YTT-C13	7	R	0.00201893	± 28	9.28	0.36
SU-YTT-C13	8	R	0.00201885	± 33	9.24	0.37
SU-YTT-C13	9	R	0.00201866	± 28	9.15	0.36
SU-YTT-C13	10	R	0.00201752	± 32	8.58	0.36
SU-YTT-C13	11	R	0.00201911	± 30	9.37	0.36
SU-YTT-C13	12	R	0.00201765	± 36	8.64	0.37
SU-YTT-C13	13	R	0.00201730	± 38	8.46	0.38
SU-YTT-C13	14	R	0.00201747	± 39	8.55	0.38
SU-YTT-C13	15	R	0.00201826	± 29	8.94	0.36
SU-YTT-C13	16	R	0.00201717	± 34	8.40	0.37
SU-YTT-C13	17	C	0.00201800	± 35	8.81	0.37
SU-YTT-C13	18	C	0.00201834	± 37	8.98	0.37
SU-YTT-C13	19	C	0.00201823	± 46	8.93	0.40
SU-YTT-C13	20	C	0.00201876	± 36	9.20	0.37
SU-YTT-C13	21	C	0.00201863	± 35	9.13	0.37
SU-YTT-C13	22	C	0.00202020	± 26	9.92	0.35
SU-YTT-C13	23	C	0.00201830	± 30	8.96	0.36
SU-YTT-C13	24	C	0.00201954	± 33	9.58	0.37

Table S3. Continued.

Sample name	Analysis point	Rim(R)/ Core(C)	Drift corrected data $^{18}O/^{16}O$		$\delta^{18}O$ quartz (‰)	Error (±1σ)
SU-YTT-C14	1	R	0.00201828	± 40	8.96	0.38
SU-YTT-C14	2	R	0.00201774	± 57	8.68	0.43
SU-YTT-C14	3	R	0.00201817	± 33	8.90	0.37
SU-YTT-C14	4	R	0.00201715	± 29	8.39	0.36
SU-YTT-C14	5	R	0.00201792	± 46	8.78	0.40
SU-YTT-C14	6	R	0.00201915	± 31	9.39	0.36
SU-YTT-C14	7	R	0.00201747	± 28	8.55	0.35
SU-YTT-C14	8	R	0.0020168	± 39	8.21	0.38
SU-YTT-C14	9	R	0.00201977	± 33	9.70	0.37
SU-YTT-C14	10	C	0.00201730	± 32	8.47	0.36
SU-YTT-C14	11	C	0.00201666	± 34	8.14	0.37
SU-YTT-C14	12	C	0.00201753	± 32	8.58	0.36
SU-YTT-C14	13	C	0.00201951	± 40	9.57	0.38
SU-YTT-C14	14	C	0.00201904	± 49	9.33	0.41
SU-YTT-C14	15	C	0.00201824	± 26	8.94	0.35
SU-YTT-C14	16	C	0.00201749	± 37	8.56	0.38
SU-YTT-C14	17	C	0.00201676	± 29	8.20	0.36
SU-YTT-C14	18	C	0.00201823	± 37	8.93	0.37
SU-YTT-C14	19	C	0.00201750	± 37	8.56	0.37
SU-YTT-C14	20	C	0.00201967	± 35	9.65	0.37
SU-YTT-C15	1	R	0.00201840	± 43	9.01	0.39
SU-YTT-C15	2	R	0.00201927	± 34	9.45	0.37
SU-YTT-C15	3	R	0.00201852	± 43	9.08	0.39
SU-YTT-C15	4	R	0.00201911	± 34	9.37	0.37
SU-YTT-C15	5	R	0.00201985	± 36	9.74	0.37
SU-YTT-C15	6	C	0.00201930	± 28	9.47	0.36
SU-YTT-C15	7	C	0.00201898	± 40	9.31	0.38
SU-YTT-C15	8	C	0.00201904	± 21	9.33	0.34
